# NGS based identification of mutational hotspots for targeted therapy in anaplastic thyroid carcinoma

**DOI:** 10.18632/oncotarget.17300

**Published:** 2017-04-20

**Authors:** Vera Tiedje, Saskia Ting, Thomas Herold, Sarah Synoracki, Soeren Latteyer, Lars C. Moeller, Denise Zwanziger, Martin Stuschke, Dagmar Fuehrer, Kurt Werner Schmid

**Affiliations:** ^1^ Department of Endocrinology and Metabolism, Endocrine Tumour Center at West German Cancer Center, University Hospital Essen, University of Duisburg-Essen, Duisburg-Essen, Germany; ^2^ Institute of Pathology, University Hospital Essen, University of Duisburg-Essen, Duisburg-Essen, Germany; ^3^ German Cancer Consortium (DKTK), German Cancer Research Center (DKFZ), Heidelberg, Germany; ^4^ Department of Radiotherapy, West German Cancer Center, University Hospital Essen, University of Duisburg-Essen, Duisburg-Essen, Germany

**Keywords:** next generation sequencing, anaplastic thyroid carcinoma, targeted therapy

## Abstract

**Context:**

Anaplastic thyroid carcinoma (ATC) represents one of the most aggressive carcinomas with no consistent survival benefit when treated with conventional radiochemotherapy. Approaches targeting “oncogene addiction” of ATC are increasingly explored and first promising results have been reported in single case studies.

**Objective:**

To determine the prevalence of mutations in known thyroid oncogenes and signalling pathways amendable to targeted therapy in a large cohort of ATC.

**Results:**

In 118 ATC (57 male/ 61 female) a total of 165 mutations were found. Genes involved in the MAPK/ERK and PI3K pathway (*BRAF* 11.0%, *HRA*S 4.2%, *KRAS* 7.6%, *NRAS* 7.6%, *PI3KC*A 11.8%) were altered in 33%. Targetable receptor tyrosine kinases were mutated in 11%. The most frequently altered genes were TERT in 86/118 (73%) and p53 in 65/118 (55%) cases. No mutations were found analysing *ALK*, *KIT*, *MET* and *mTOR*.

**Materials and Methods:**

Next generation sequencing (NGS) was performed in FFPE samples from 118 ATC using MiSeq (Illumina) and CLC Cancer Research Workbench (CLCbio; Qiagen) for mutation analysis in: *ALK*, *BRAF*, *CDKN2A*, *EGFR*, *ERBB2*, *HRAS*, *KIT*, *KRAS*, *MET*, *mTOR*, *NRAS*, *PDGFRA*, *PI3KCA*, *p53*, *RB1*, *RET* and *TSC2*. Sanger sequencing was used to detect *TERT* promotor mutations.

**Conclusions:**

To our knowledge this is the largest study analysing mutations for targeted therapy of ATC. We found that 33% of ATC harbour mutations in pathways amendable to targeted therapy. Molecular screening in ATC is suggested for targeted therapies since current conventional treatment for ATC proved mainly futile.

## INTRODUCTION

Anaplastic thyroid carcinoma (ATC) represents one of the most aggressive human malignancies [1]. Treatment of ATC is challenging, since it is an orphan disease and no established therapies offering a survival benefit are available. Thus prognosis remains very poor with a median survival of only 6 months [1].

Recently targeted therapies have been increasingly explored in ATC, based on the concept that molecular drivers of ATC may represent promising targets to be efficiently tackled. Genetic alterations in ATC enfold genes involved in the PI3K and MAPK/ERK pathway, receptor tyrosine kinases and tumour suppressor genes [2–5]. Activation of the PI3K cascade has been considered a hallmark for dedifferentiation of differentiated thyroid carcinoma (DTC) since activating mutations in this cascade are found with increasing prevalence in ATC compared to DTC [6], reaching 9–18% frequency for *PI3KCA* in ATC [5, 7]. Gene alterations in the MAPK/ERK pathway, e.g. *BRAF* mutations, have been identified in around 60% of papillary thyroid carcinoma (PTC) [8] and 15–45% of ATC [7, 9].

The most frequently mutated gene in ATC is *p53* [7]. Mutations in *p53* cause protein inactivation leading to inactivation of apoptosis and cell cycle progression [10]. Alterations of p53 are very rare in DTC, consistent with a late event in tumour dedifferentiation [11]. Knowledge of the genetic background of ATC has been broadened by the discovery of two human telomerase reverse transcriptase (*TERT*) mutations, the C228T and the C250T alterations, conferring increased telomerase activity [12]. These mutations have been suggested to further contribute to ATC aggressiveness [9]. Furthermore the group of Xing M *et al*. showed that coexisting *BRAF* V600E and *TERT* C228T alterations in papillary thyroid carcinoma (PTC) are associated with a significantly higher risk of recurrence and poorer outcome as well as older age and distant metastasis in ATC [9, 13].

Since the first identification of driver mutations in ATC, several case reports have shown response to targeted therapy in ATC patients. These included the application of inhibitors to *BRAF* [14–16], *mTOR* [17] and *ALK* [18]. In these patients the tumour mass reduction was related to the demonstration of the putative driver mutation followed by the consecutive targeted therapy. On the other hand, the multikinase-inhibitors sorafenib and pazobanib have failed to demonstrate survival benefit; however, it has to be emphasized that deductions from these studies are limited due to evaluation of only 20 and 15 ATC patients, respectively [19, 20]. In contrast axitinib, a VEGFR 1-3 inhibitor, was reported to confer disease stabilization over a period of 12 months in 2 patients [21]. In a phase II trial evaluating the EGFR inhibitor gefitinib disease stabilization was reported in 5 of 25 ATC patients [22].

Over the past 2 years, several groups have applied high throughput sequencing either by next generation sequencing (NGS) or whole genome sequencing to unravel the molecular signature of ATC. So far however, these studies were performed on ATC series comprising no more than 33 samples [7]. Hence, the prevalence of genetic alterations representing possible targets for ATC therapy that could be approached in clinical practice is still unknown. Furthermore, it remains particularly unclear whether screening for a set of mutations may help to define a subset of ATC patients, who could benefit from targeted therapy. These limitations in mind, we set out to comprehensively determine the prevalence of mutations in distinguished thyroid oncogenes and signalling pathways currently amendable to targeted drug therapy in a large cohort of ATC.

## RESULTS

In this study the paraffin embedded tumour tissue of 118 ATC patients (57 males/61 females) with a median age at diagnosis of 65 years (ranging from 26 to 90 years) were analysed for mutations in hotspots of classical oncogenes. Patients characteristics are listed in Table 1.

**Table 1:** Patients characteristics

Number of tumours	118
Female	57
Male	61
Derived from DTC*	35
Derived from PDTC*	4
*de novo* ATC***	64
Age at diagnosis [years]	
median	65
range	26–90

*DTC: Differentiated Thyroid Carcinoma. ^*^PDTC: Poorly Differentiated Thyroid Carcinoma. ^**^*ATC: Anaplastic Thyroid Carcinoma.

Mutation in the *TERT* promoter hotspots C250T and C228T were the most frequent alterations found in 86/118 (73%) tumour specimens, followed by p53 alterations in 65/118 (55%), *RAS* (*HRAS*, *KRAS* and *NRAS*) in 23/118 (19.5%), *CDKN2A* mutations in 20/118 (16.9%), *PI3KCA* in 14/118 (11.8%), *BRAF* in 13/118 (11%) and *RET* in 9/118 (7.6%) of ATC. *EGFR*, *ERBB2*, *PDGFRA*, *RB1* and *TSC2* were mutated in less than 2% of all ATC. In *ALK*, *KIT* and *MET* no mutations were found in the investigated exons. Identified mutations are listed in Supplementary Table 1.

Mutations in classical thyroid oncogenes e.g. *BRAF*, *PI3KCA* and *RAS* were detected in 40/118 (33%) ATCs. Genetic variations in the *BRAF* gene were detected in 13 of 118 (11%) ATCs. In 9 ATCs the classical V600E mutation was present, while the G469A variation was detected in 2 tumours, and the G469V and the D594A mutation were found in 1 ATC each.

Genetic alterations in *PI3KCA* were detected in 14/118 (11.8%) ATCs and comprised the E545K variation in 10 tumours, the H1047R variation in 3 tumours and the N1044L variation in 1 tumour.

Genetic alterations in the *HRAS*, *KRAS* and *NRAS* genes were found in 23/118 (19.5%) of all ATCs. In *HRAS* the Q61R mutation was detected in 4/118 (3.4%) and the G60S mutation in 1/118 (0.9%) tumours. In *KRAS* 3 distinct mutations were identified: G12R in 7 (5.9%), H27L and G12V each in 1 (0.9%) ATC sample. In *NRAS*, the variation Q61R was found in 7 and the G61K mutation was detected in 2 ATCs.

**Figure 1:**
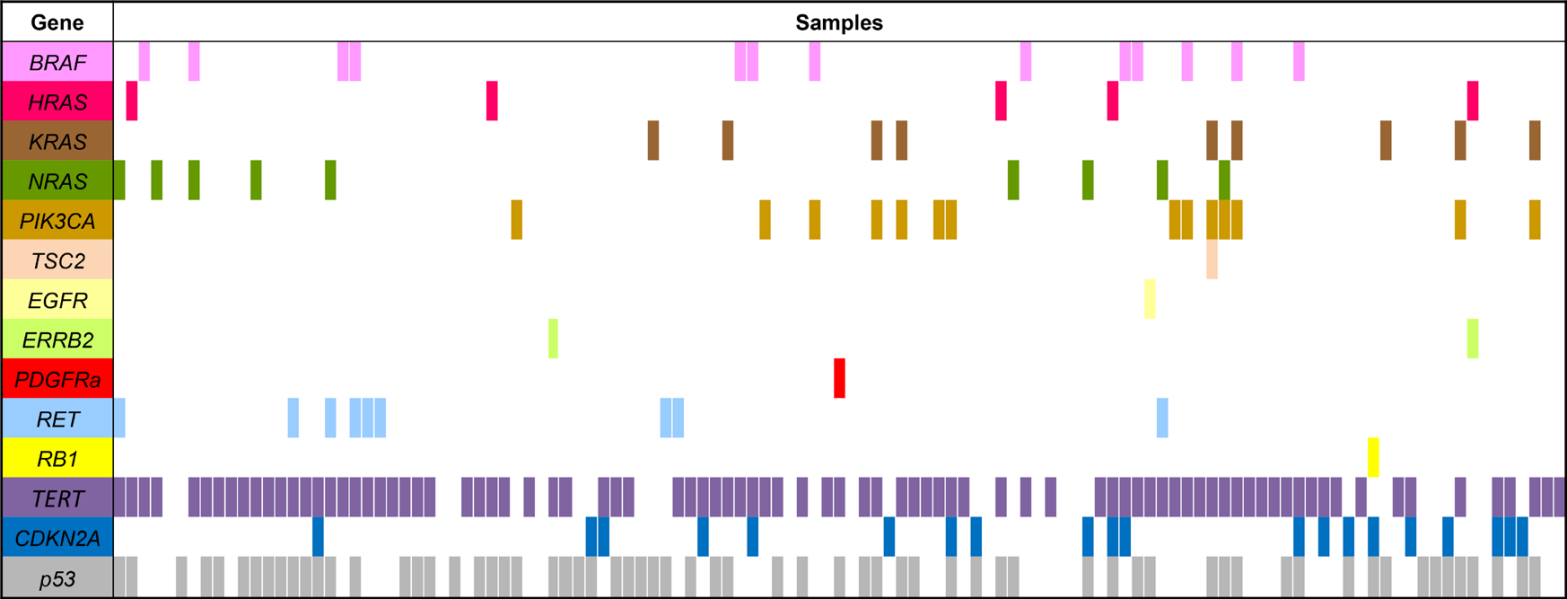
Mutations in genes that can be targeted by molecular drug therapy in 118 ATC primary tumour samples P53 and TERT mutations were analyzed as a hallmark of ATC.

Only 8 of 40 ATCs harboured single mutational events in the MAPK/ERK- and PI3K-pathways: 4 *BRAF* (two V600E, G469V and G469A), 3 *PI3KCA* (two E545K and H1047R) and 1 *NRAS* (Q61K) variation. In 11 ATCs concurrent mutations in *BRAF*, *PI3KCA* and *RAS* were found: 7 ATCs showed *PI3KCA* and *RAS* mutations, 2 *BRAF* and *PI3KCA* variations, 1 *BRAF* and *RAS* and 1 ATC had mutation in *BRAF*, *PI3KCA* and *RAS*. In the other 21 ATCs concurrent *p53* and/or *CDKN2A* mutations or *RET* or *ERBB2* mutations were detected (Figure 1).

Mutations in the receptor tyrosine kinases *EGFR*, *PDGFRA*, *ERBB2* and *RET* were identified in 13 ATC and involved *EGFR* (T790M, *N* = 1), *PDGFRA* (S667P, *N* = 1) and *ERBB2* (L787P and G746S, *N* = 2). Variations in *RET* were detected in 9/118 (7.6%) ATC: 4 ATC with Y791F, 2 ATC with P596L and 1 ATC each with E818K or E595K.

Mutations in the *TERT* promotor (C250T and C228T) were detected in 73% of ATC. The C228T mutation was far more frequent and occurred in 82/118 compared to the C250T mutation in only 3/118 tumours. In 1 ATC sample both mutations were found by Sanger sequencing (Supplementary Table 1).

The tumour suppressor gene p53 was mutated in 65/118 (55%) tumour samples and in total 77 mutations occurred: R248Q in 7 ATCs; T125A, R209fs, P278S and R280T in 5 ATCs; S183* and R273C in 3 ATCs each; and C135F, Y220C, G266E, V272L, R282W and E285K in 2 ATCs each (Supplementary Table 1).

For the cell cycle regulating gene *CDKN2A* the most frequent mutation was the E88G missense mutation, detected in 8/118 (7%) of ATC samples. Further *CDKN2A* mutations were L65P, D84G and R98Q (in 2 ATCs each); and G83R, P72L and H66R in 1 ATC 1 each (Supplementary Table 1).

In 10/118 (8.5%) ATC no mutations were found in the analyzed genes. In 44/118 (37.3%) only one gene was altered: *BRAF* (V600E, G469V and G469A) in four ATC, *PI3KCA* and *RET* in three ATC each, *NRAS* in one ATC and *p53* in 27 ATC.

Furthermore no *ALK*, *KIT*, *MET* or *mTOR* mutations were detected in the sequenced exons of the ATC series.

In ATC harbouring residues of DTC, *BRAF* and *PI3KCA* mutations were more prevalent than in *de novo* ATC samples (22.9% versus 6.3% and 22.9% versus 6.3%). As for TERT and p53 variations described as hallmarks in thyroid cancer dedifferentiation, only p53 mutation prevalence was higher in *de novo* ATC in comparison to ATC derived DTC (64.1% versus 34.3%).

## DISCUSSION

Conventional radiochemotherapy in ATC does mostly not prolong survival, while single case reports have shown impressive tumour shrinkage after targeted drugs inhibiting single mutations in ATC [14, 17, 18]. Aiming to develop novel treatment strategies for ATC and to improve outcome, we have performed next generation sequencing of a large ATC series to clarify which mutational hotspots and/or signalling pathways are affected and may thus be considered for targeted drug therapy.

We found genetic alterations in the classical oncogenes (*BRAF*, *RAS* and *PIK3CA*) involved in thyroid tumourigenesis in 33% (40/118) of all ATC. Moreover, in 11% (13/118) of all ATC, mutations in targetable receptor tyrosine kinases (e.g. *RET*) were detected. In 40% (47/118) of all ATC mutations involved in the MAPK/ERK or PI3K pathways or receptor tyrosine kinase encoding genes were altered.

*BRAF* V600E is probably the most studied mutation in PTC and ATC. In our cohort a lower prevalence of *BRAF* V600E was detected than reported in the literature. Kunstman *et al*. [5] found a prevalence of 27% *BRAF* V600E mutations in their series and Landa *et al*. [7] detected mutations even in 45% of the investigated ATC samples. Several reasons may be considered: First the analysed cohorts by Kunstman and Landa only included 21 [5] and 33 ATC samples [7] and therefore the prevalence of BRAF mutation in these cohorts might be overrated. Secondly, ATC in our series may have developed *de novo* rather than evolved by dedifferentiation from DTC or PDTC. Hence we retrieved data from ATC primary report and found that only 29.6% of our samples had indication of concurrent or preceding DTC. In previous studies between 40–45% tumour specimens had concurrent DTC [5, 7].

Interestingly, besides *BRAF* V600E, 3 other *BRAF* variants were detected in our ATC series: G469V, G469A and D594A. These alterations are known oncogenes in malignant melanoma [23, 24] and non-small-cell lung cancer (NSCLC) [25, 26] but have so far not been reported in thyroid carcinoma. Since they were found in other human malignancies, we consider these alterations as likely oncogene also in the context of ATC.

In single case reports, ATC patients with *BRAF* V600E mutated tumours were treated with a BRAF-inhibitor [14–16]. According to our analysis, targeted BRAF treatment would only be an option in few of our ATC patients.

*PIK3CA* alterations have been reported in 4.5% [5] and 18% [7] of ATC using NGS. We found *PI3KCA* mutations in 11.8 % of our ATC samples, in particular the E545K *PI3KCA* alteration in the helical domain and the H1047R variation located in the kinase domain. Earlier reports identified the same mutations [5, 7] in ATC samples. Small molecule PI3K-inhibitors are approved for the treatment of lymphomas and chronic lymphatic leukaemia and are currently evaluated in solid tumours e.g. breast cancer [27] and NSCLC [28]. In these malignancies, activation of the PI3K-pathway (*PI3KCA* mutations or *PTEN* loss) was consistent with good response to PI3K-inhibitors [28, 29]. However, in a phase II study including glioblastoma patients no association between pathway activation in tumour tissues and efficacy of PI3K-inhibitor treatment was found [28]. The efficacy of PI3K-inhibitors in ATC has not been investigated in the clinical setting so far. However, since almost 12% of our ATC samples harboured activating PI3K cascade mutation in agreement with previous studies by Landa *et al*. [7] and Xing *et al*. [30] showing that activation of PI3K signalling is highly prevalent in ATC, we suggest that PI3K inhibitors could be worthwhile exploring in clinical studies.

*RAS* mutations were detected in 23 ATCs: Among them are mutations that have frequently been found in thyroid tumours [31]. The *HRAS* G60S and *KRAS* H27L found in 1 ATC each, have so far not been linked to thyroid cancer. *RAS* mutations are known events in benign and malignant thyroid tumours [32, 33]. In PTC they were shown to be mutually exclusive with BRAF mutations and are consistent with follicular variant PTC [11], but in ATC *RAS* mutations may occur concurrently with other mutations including *BRAF* V600E [7]. In *RAS*-mutated PTC the RAF-MEK-ERK cascade has been shown to confer cell proliferation, however with smaller transcriptional output than *BRAF* mutations [11]. This is explained by an ERK-induced negative feedback that disrupts *RAF* dimerization [34]. In *RAS* mutated ATC this feedback is mostly not observed [7] which could potentially render ATC with activating *RAS* mutations susceptible to downstream MEK-Inhibitors.

Murugan and Xing *et al*. [35] were the first to describe mutations in *ALK* exons 20 and 23 in 18 ATC. In our series no *ALK* mutations were detected in exons 20–25. In a recent report of our group [36], *ALK* mutations were exclusively detected in exons 1, 3, 16, 28 and 29, which were not addressed in the present NGS approach, suggesting that probably the entire ALK gene has to be sequenced to assess for genetic alterations in ATC.

We also analysed genetic variations in receptor tyrosine kinases *EGFR*, *RET*, *PDGFRA* and *ERBB2* since targeted therapies for these tyrosine kinases are available for human malignancies. The *EGFR* T790M variation is well studied in lung cancer and is known to confer EGFR-inhibitor resistance [37]. In our ATC series *EGFR* mutations were shown to be rare events and in clinical practice mutational screening would be helpful only when treatment with an EGFR-inhibitor is planned.

Hereditary medullary thyroid carcinoma (MTC) is characterized by germline *RET* mutations [38] and in 40–60% of sporadic MTC somatic *RET* mutations are present [39, 40], which cause constitutive activation of the RET tyrosinkinase and in consequence MAPK/ERK and PI3K pathway activation. In follicular-cell derived thyroid carcinoma *RET* mutations are generally rare events [41]. In a previous report *RET* gene variations were found in 1/22 (4.5%) ATC investigated [5]. In our study we found *RET* mutations in 9/118 (7.6%) ATC: in 4 ATC the Y791F variation was detected, which is described in Multiple endocrine neoplasia type 2A (MEN2A) cases, but is interestingly not associated with MTC development [42]. The P596L, E818K (in 2 ATCs each) and the E595K *RET* variation (1 ATC) have so far not been described in association with thyroid cancer or other human malignancies and their biological relevance is not known.

Genetic variations in *ERBB2* and *PDGFRA* were rare events (2/118 and 1/118). This is in accordance with previous reports [5, 7] and hence screening does not seem to be warranted in ATC.

Genetic variations in tumour suppressor genes are frequent events in cancer. Inactivating *p53* mutations are a molecular hallmark of ATC. In agreement within this and recent large-scale analysis of ATC (5, 7) *p53* was frequenty mutated in our cohort with genetic variations in 65 of 118 tumours. Somatic *p53* mutations are very rare in DTC and it has been shown in mouse models that p53 inactivation contributes to the progression from PTC to ATC [43]. Less well studied are other genetic variations involved in cell cycle regulation like mutations in the cyclin-dependent kinases (CDK). Kunstman *et al*. reported single mutations in the *CDKN1B* and *CDKN2C* encoding genes [5]. In our study we found *CDKN2A* mutations in 23 ATC (19.5%). Thereby, the most frequent mutation was the E88G variation, detected in 14 ATC, which has not previously been described in ATC. Reports have shown that co-targeting CDK 4/6 and BRAF is efficient in *BRAF* mutated tumours to overcome BRAF-inhibitor resistance [44].

Weaknesses of our study are the lack of clinical data, rendering correlation of the genetic findings with disease course impossible. Furthermore, only hotspot regions of genes were sequenced, hence we cannot exclude that mutational events were present in other exons. The strength of our study is the application of a NGS which is already routinely applied in patient care at the Essen University Hospital's Comprehensive Cancer Centre underlining its applicability in clinical practice. Moreover, our series presents the largest ATC cohort comprehensively analysed for mutations in targetable oncogenes so far and all tissues were classified as ATC by board certified pathologists of the German Thyroid Pathology Reference Centre at the University Hospital of Essen.

In summary we show, that mutations in hotspots of targetable oncogenes are present in approximately 33% of ATC. We therefore propose a panel of oncogenes (BRAF, PI3KCA and RAS) that should be screened in clinical practice in patients with newly discovered ATC in order to offer patients targeted therapy treatment as part of a highly individualized treatment approach where feasible and if desired by the patient. The clinical care of ATC patients urgently needs to be improved including central pathology review, national and international networks and accessibility to basket studies testing novel targeted and other therapies.

## MATERIALS AND METHODS

We retrospectively analyzed a study cohort of 118 patients with ATC. FFPE tissue from primary tumour was used to perform NGS. All samples were reviewed by two board-certified pathologists and diagnosis was made according to the WHO classification [45]. When DTC co-occurred in tumour specimen, the respective ATC case was regarded to be derived from DTC.

After deparaffinization of FFPE tissue with deparaffinization solution (Qiagen, USA) and incubation with 20 μl proteinase K solution (20 mg/ml) over night at 56°C, DNA isolation from the primary tumour tissue was performed using QIAamp DNA FFPE tissue kit (Qiagen, USA) according to the manufacturer's instructions. DNA concentrations were determined by Qubit^®^ 2.0 Fluorometer dsDNA HS assay kit (LifeTechnologies, USA). All samples showed a concentration between 1–100 ng/μl.

A total amount of 45 ng DNA was used to perform multiplex-pcr (four primer pools with 10 ng/primer pool + 10% excess). Multiplex PCR and purification were performed with the GeneRead DNAseq Custom Panel V2, GeneRead DNAseq Panel PCR Kit V2 (Qiagen, USA) and Agencourt^®^ AMPure^®^ XP Beads (Beckmann, USA), followed by measurement of total DNA amount by Qubit^®^ 2.0 Fluorometer dsDNA HS assay kit. The library preparation was performed with NEBNext Ultra DNA Library Prep Set for Illumina (New England Biolabs, USA), according to the manufacturer's recommendations by using 24 different indices per run. The pooled library was sequenced on MiSeq (Illumina; 2 × 150 bases paired-end run) and analyzed by Cancer Research Workbench (CLC Bio, Qiagen, USA).

For targeted sequencing a customized ATC-panel was designed containing regions of interest. The ATC-panel contains hot-spot regions out of 17 genes. The analyzed exons are listed in Supplementary Table 2. The regions were covered by a total of 136 amplicons. In all runs an average coverage of approximately 8000 × was obtained.

Within CLC Cancer Research Workbench demultiplexed paired-end sequencing data was mapped to human genome (version hg19). A local realignment was performed to reach better quality (especially for regions with small insertions or deletions). All reads, which were mapped outside of targeted-regions, got deleted after mapping process. Within target regions information from different databases (Cosmic, Clinvar, dbSNP, 1000 genome project, HapMap) was annotated.

Performing a second filtering-step all reference-variants and variants found in dbSNP common, 1000 genome project and HapMap were deleted. All variants found in Cosmic, Clinvar or those which weren't annotated in any database were listed. Therefore, an allele-frequency of minimum 5% and coverage of at least 100 mapped-reads were the selection-parameters.

For detection of the two known TERT promotor mutations on chromosome 5 position g.1295250C > T and g.1295228C > T (reference genome hg19) Sanger sequencing was performed. The promoter region of TERT was amplified by PCR, followed by direct sequencing. PCR reaction was carried out with oligonucleotides TERT fwd (CCTGCCCCTTCACCTTCCAG) and TERT rev (AGGACGCAGCGCTGCCTGAA) in a total volume of 50 μl with HotStar Taq Mastermix (Qiagen) according to the recommendations of the supplier. Amplification was performed in a Biometra T3000 Thermocycler (Biometra, Göttingen) with 10 min of initial enzyme activation at 95°C followed by 45 cycles of denaturation at 94°C for 30 sec, oligonucleotide annealing at 62°C for 60 sec and extension at 72°C for 30 sec. Sequences of both, forward and reverse strand were analyzed on an ABI 3500 Genetic Analyser (LifeTechnologies) using BigDye Terminator chemistry v3.1 (LifeTechnologies).

## SUPPLEMENTARY TABLES





## Abbreviations

ATC: Anaplastic Thyroid Carcinoma
DTC: Differentiated Thyroid Carcinoma
MTC: Medullary Thyroid Carcinoma
NGS: Next Generation Sequencing
NSCLC: Non-small Cell Lung Cancer
PTC: Papillary Thyroid Carcinoma
TERT: Telomerase Reverse Transcriptase
