# Supplementary material for: NGS based identification of mutational hotspots for targeted therapy in anaplastic thyroid carcinoma
Source: Oncotarget. 2017 Apr 20;8(26):42613–20. doi: 10.18632/oncotarget.17300 (PMC5522092; doi:10.18632/oncotarget.17300)
Supplement: Supplementary file 1 [file oncotarget-08-42613-s001.pdf]

NGS based identification of mutational hotspots for targeted therapy in anaplastic thyroid carcinoma

Supplementary Materials

Supplementary Table 1: Mutations in the analysed genes 118 ATC primary tumour samples and the relative prevalence of each mutation in %. See Supplementary_Table_1

Supplementary Table 2: Panel of analyzed genes and exons

Gene	Exon
ALK	20–25
BRAF	11, 15
CDKN2A	2
EGFR	18–21
ERBB2	20, 23
HRAS	2–4
KRAS	2–4
KIT	9–11, 13, 17, 18
MET	14
mTOR	4, 5
NRAS	2–4
PDGFRA	12, 14, 18
PIK3CA	10, 21
RB1	18
RET	10, 11, 13–16
TSC2	30
p53	4–9
