# Supplementary material for: NGS based identification of mutational hotspots for targeted therapy in anaplastic thyroid carcinoma
Source: Oncotarget. 2017 Apr 20;8(26):42613–20. doi: 10.18632/oncotarget.17300 (PMC5522092; doi:10.18632/oncotarget.17300)
Supplement: Supplementary file 2 [file oncotarget-08-42613-s002.docx]

| **Sample** | ***BRAF*** | ***CDKN2A*** | ***EGFR*** | ***ERRB2*** | ***HRAS*** | ***KRAS*** | ***NRAS*** | ***PDGFRA*** | ***PIK3CA*** | ***RB1*** | ***RET*** | ***TERT*** | ***TP53*** | ***TSC2*** |
| --- | --- | --- | --- | --- | --- | --- | --- | --- | --- | --- | --- | --- | --- | --- |
| **1** |  |  |  |  |  |  | Q61R  21.39% |  |  |  | E595L  12.77% | C228T | V272Q  41.75% |  |
| **2** |  |  |  |  | Q61R  50.98% |  |  |  |  |  |  | C228T | H179L  49.54% |  |
| **3** | G469V  21.73% |  |  |  |  |  |  |  |  |  |  | C228T |  |  |
| **4** |  |  |  |  |  |  | Q61L  8.85% |  |  |  |  | C228T |  |  |
| **5** |  |  |  |  |  |  |  |  |  |  |  |  |  |  |
| **6** |  |  |  |  |  |  |  |  |  |  |  |  | Q285V  26.88% |  |
| **7** | D594A  22.12% |  |  |  |  |  | Q61L  23.52% |  |  |  |  | C228T |  |  |
| **8** |  |  |  |  |  |  |  |  |  |  |  | C228T | Y163C  9.16% |  |
| **9** |  |  |  |  |  |  |  |  |  |  |  | C228T | C135F  60.70% |  |
| **10** |  |  |  |  |  |  |  |  |  |  |  | C228T |  |  |
| **11** |  |  |  |  |  |  |  |  |  |  |  | C250T | Y234N  35.92% |  |
| **12** |  |  |  |  |  |  | Q61R  28.11% |  |  |  |  | C228T | G245D  61.85% |  |
| **13** |  |  |  |  |  |  |  |  |  |  |  | C250T | F212fs  27.96% |  |
| **14** |  |  |  |  |  |  |  |  |  |  |  | C228T | R248Q  56.12% |  |
| **15** |  |  |  |  |  |  |  |  |  |  | T791F  6.34% | C228T | R209fs  53.93% |  |
| **16** |  |  |  |  |  |  |  |  |  |  |  | C228T | P278S  31.44% |  |
| **17** |  | L65P  58.77% |  |  |  |  |  |  |  |  |  | C228T | P278S  82.31% |  |
| **18** |  |  |  |  |  |  | Q61R  23.37% |  |  |  | E818L  5.56% | C228T | R209fs  53.93% |  |
| **Sample** | ***BRAF*** | ***CDKN2A*** | ***EGFR*** | ***ERRB2*** | ***HRAS*** | ***KRAS*** | ***NRAS*** | ***PDGFRA*** | ***PIK3CA*** | ***RB1*** | ***RET*** | ***TERT*** | ***TP53*** | ***TSC2*** |
| **19** | V600E  28.35% |  |  |  |  |  |  |  |  |  |  | C228T |  |  |
| **20** | V600E  7.78% |  |  |  |  |  |  |  |  |  | T791F  6.44% | C228T | P80L  8.65% |  |
|  |  |  |  |  |  |  |  |  |  |  |  | C250T |  |  |
| **21** |  |  |  |  |  |  |  |  |  |  | T791F  50.91% | C228T |  |  |
| **22** |  |  |  |  |  |  |  |  |  |  | Q818L  6.9% | C228T |  |  |
| **23** |  |  |  |  |  |  |  |  |  |  |  | C228T |  |  |
| **24** |  |  |  |  |  |  |  |  |  |  |  | C228T | R248Q  20.18% |  |
| **25** |  |  |  |  |  |  |  |  |  |  |  | C228T | G266E  20.79% |  |
| **26** |  |  |  |  |  |  |  |  |  |  |  | C228T | H179Y  22.52% |  |
| **27** |  |  |  |  |  |  |  |  |  |  |  |  |  |  |
| **28** |  |  |  |  |  |  |  |  |  |  |  |  | R209fs  59.25% |  |
| **29** |  |  |  |  |  |  |  |  |  |  |  | C228T |  |  |
| **30** |  |  |  |  |  |  |  |  |  |  |  | C228T | R213*  15.32% |  |
| **31** |  |  |  |  | Q61R  38.52% |  |  |  |  |  |  | C228T | V272L  32.08% |  |
| **32** |  |  |  |  |  |  |  |  |  |  |  | C228T | G266E |  |
| **33** |  |  |  |  |  |  |  |  | N1044L  28.99% |  |  |  | P278S  15.07% |  |
| **34** |  |  |  |  |  |  |  |  |  |  |  | C228T |  |  |
| **35** |  |  |  |  |  |  |  |  |  |  |  |  |  |  |

| **Sample** | ***BRAF*** | ***CDKN2A*** | ***EGFR*** | ***ERRB2*** | ***HRAS*** | ***KRAS*** | ***NRAS*** | ***PDGFRA*** | ***PIK3CA*** | ***RB1*** | ***RET*** | ***TERT*** | ***TP53*** | ***TSC2*** |
| --- | --- | --- | --- | --- | --- | --- | --- | --- | --- | --- | --- | --- | --- | --- |
| **36** |  |  |  | G746S  5.16% |  |  |  |  |  |  |  | C228T | P278S  5.25% |  |
|  |  |  |  |  |  |  |  |  |  |  |  |  | P76L  6.8% |  |
| **37** |  |  |  |  |  |  |  |  |  |  |  | C228T | R209fs  9.18% |  |
| **38** |  |  |  |  |  |  |  |  |  |  |  |  | R273C  75.88% |  |
| **39** |  | R98Q  91.07% |  |  |  |  |  |  |  |  |  |  | G601Q  14.57% |  |
| **40** |  | R98Q  9.89% |  |  |  |  |  |  |  |  |  | C228T |  |  |
| **41** |  |  |  |  |  |  |  |  |  |  |  | C228T | P153fs  6.06% |  |
| **42** |  |  |  |  |  |  |  |  |  |  |  | C228T | Y220C  15.98% |  |
| **43** |  |  |  |  |  |  |  |  |  |  |  |  | R273C  12.16% |  |
|  |  |  |  |  |  |  |  |  |  |  |  |  | R248Q  9.07% |  |
|  |  |  |  |  |  |  |  |  |  |  |  |  | C238Y  19.01% |  |
| **44** |  |  |  |  |  | G12V  40.32% |  |  |  |  |  |  | R273C  57.04% |  |
| **45** |  |  |  |  |  |  |  |  |  |  | T791F  5.58% |  | P278S  16.86% |  |
| **46** |  |  |  |  |  |  |  |  |  |  | P596L  5.59% | C228T |  |  |

| **Sample** | ***BRAF*** | ***CDKN2A*** | ***EGFR*** | ***ERRB2*** | ***HRAS*** | ***KRAS*** | ***NRAS*** | ***PDGFRA*** | ***PIK3CA*** | ***RB1*** | ***RET*** | ***TERT*** | ***TP53*** | ***TSC2*** |
| --- | --- | --- | --- | --- | --- | --- | --- | --- | --- | --- | --- | --- | --- | --- |
| **47** |  |  |  |  |  |  |  |  |  |  |  | C228T | P301S  6.70% |  |
|  |  |  |  |  |  |  |  |  |  |  |  |  | R248Q  13.83% |  |
|  |  |  |  |  |  |  |  |  |  |  |  |  | A161V  8.70% |  |
|  |  |  |  |  |  |  |  |  |  |  |  |  | K132R  22.30% |  |
| **48** |  |  |  |  |  |  |  |  |  |  |  |  |  |  |
| **49** |  |  |  |  |  |  |  |  |  |  |  | C228T |  |  |
| **50** |  | E88G  4.62% |  |  |  |  |  |  |  |  |  | C228T |  |  |
| **51** |  |  |  |  |  |  |  |  |  |  |  | C228T | Y220C  72.35% |  |
| **52** |  |  |  |  |  | G12R  4.33% |  |  |  |  |  | C228T | R280T  5.9% |  |
| **53** | G469A  32.08% |  |  |  |  |  |  |  |  |  |  | C228T |  |  |
| **54** | G469A  41.18% | E88G  5.84% |  |  |  |  |  |  |  |  |  | C228T |  |  |
| **55** |  |  |  |  |  |  |  |  | E545L  24.50% |  |  | C228T |  |  |
| **56** |  |  |  |  |  |  |  |  |  |  |  | C228T | R248Q  60.07% |  |
| **57** |  |  |  |  |  |  |  |  |  |  |  |  |  |  |
| **58** |  |  |  |  |  |  |  |  |  |  |  | C228T | L320*  13.42% |  |
| **59** | V600E  24.92% |  |  |  |  |  |  |  | E545L  22.82% |  |  |  |  |  |
| **60** |  |  |  |  |  |  |  |  |  |  |  | C228T |  |  |
| **61** |  |  |  |  |  |  |  | S667P  5.40% |  |  |  | C228T | T125A  7.81% |  |
| **Sample** | ***BRAF*** | ***CDKN2A*** | ***EGFR*** | ***ERRB2*** | ***HRAS*** | ***KRAS*** | ***NRAS*** | ***PDGFRA*** | ***PIK3CA*** | ***RB1*** | ***RET*** | ***TERT*** | ***TP53*** | ***TSC2*** |
| **62** |  |  |  |  |  |  |  |  |  |  |  |  |  |  |
| **63** |  |  |  |  |  |  |  |  |  |  |  | C228T | R282W  17.78% |  |
| **64** |  |  |  |  |  | G12R  19.85% |  |  | E545L  17.97% |  |  | C228T | R280T  31.47% |  |
| **65** |  | E88G  6.76% |  |  |  |  |  |  |  |  |  |  |  |  |
| **66** |  |  |  |  |  | G12R  66.58% |  |  | E545L  59.95% |  |  | C228T | R280T  69.53% |  |
| **67** |  |  |  |  |  |  |  |  |  |  |  | C228T | S183*  74.06% |  |
| **68** |  |  |  |  |  |  |  |  |  |  |  | C228T |  |  |
| **70** |  |  |  |  |  |  |  |  | E545L  7.64% |  |  | C228T |  |  |
| **71** |  | E88G  7.17% |  |  |  |  |  |  | E545L  6.32% |  |  | C228T | C176R  5.16% |  |
| **72** |  |  |  |  |  |  |  |  |  |  |  | C228T |  |  |
| **74** |  | E88G  8.80% |  |  |  |  |  |  |  |  |  |  | V157A  5.67% |  |
| **75** |  |  |  |  |  |  |  |  |  |  |  |  |  |  |
| **76** |  |  |  |  | Q61R  72.82% |  |  |  |  |  |  | C228T | R280G  72.82% |  |
| **77** |  |  |  |  |  |  | Q61R  11.66% |  |  |  |  |  | S183*  29.25% |  |
| **78** | V600E  41.51% |  |  |  |  |  |  |  |  |  |  | C228T |  |  |
| **79** |  |  |  |  |  |  |  |  |  |  |  |  |  |  |
| **80** |  |  |  |  |  |  |  |  |  |  |  | C228T |  |  |
| **81** |  |  |  |  |  |  |  |  |  |  |  |  |  |  |

| **Sample** | ***BRAF*** | ***CDKN2A*** | ***EGFR*** | ***ERRB2*** | ***HRAS*** | ***KRAS*** | ***NRAS*** | ***PDGFRA*** | ***PIK3CA*** | ***RB1*** | ***RET*** | ***TERT*** | ***TP53*** | ***TSC2*** |
| --- | --- | --- | --- | --- | --- | --- | --- | --- | --- | --- | --- | --- | --- | --- |
| **82** |  |  |  |  |  |  |  |  |  |  |  |  |  |  |
| **83** |  | E88G  11.91% |  |  |  |  | Q61R  9.35% |  |  |  |  |  | T125A  6.49% |  |
|  |  | N84G  6.53% |  |  |  |  |  |  |  |  |  |  |  |  |
| **84** |  |  |  |  |  |  |  |  |  |  |  | C228T |  |  |
| **85** |  | G83R  7.74% |  |  | G60S  6.84% |  |  |  |  |  |  | C228T | R248Q  22.34% |  |
| **86** | V600E  18.59% | P72L  6.22% |  |  |  |  |  |  |  |  |  | C228T |  |  |
| **87** | V600E  22.25% |  |  |  |  |  |  |  |  |  |  | C228T | Q100*  8.93% |  |
| **88** |  |  | T790M  5.51% |  |  |  |  |  |  |  |  | C228T | A138L  53.59% |  |
| **89** |  |  |  |  |  |  | Q61R  31.38% |  |  |  | P596L  23.01% | C228T |  |  |
| **90** |  |  |  |  |  |  |  |  | H1047R  35.81% |  |  | C228T |  |  |
| **91** | V600E  23.05% |  |  |  |  |  |  |  | E545L  12.55% |  |  | C228T |  |  |
| **92** |  |  |  |  |  |  |  |  |  |  |  | C228T |  |  |
| **93** |  |  |  |  |  | G12R  14.11% |  |  | E545L  6.78% |  |  | C228T | M237I  12.18% | R1200W  5.50% |
| **94** |  |  |  |  |  |  | Q61R  32.63% |  | H1047R  20.76% |  |  | C228T | V157G  13.14% |  |
| **95** | V600E  9.91% |  |  |  |  | G12R  26.21% |  |  | E545L  25.95% |  |  | C228T | T329I  15.03% |  |
|  |  |  |  |  |  |  |  |  |  |  |  |  | R280T  12.98% |  |
|  |  |  |  |  |  |  |  |  |  |  |  |  | E62K  8.78% |  |
| **96** |  |  |  |  |  |  |  |  |  |  |  | C228T |  |  |

| **Sample** | | ***BRAF*** | | ***CDKN2A*** | | ***EGFR*** | | ***ERRB2*** | | ***HRAS*** | | ***KRAS*** | | ***NRAS*** | | ***PDGFRA*** | | ***PIK3CA*** | | ***RB1*** | | ***RET*** | | ***TERT*** | | ***TP53*** | ***TSC2*** |  |
| --- | --- | --- | --- | --- | --- | --- | --- | --- | --- | --- | --- | --- | --- | --- | --- | --- | --- | --- | --- | --- | --- | --- | --- | --- | --- | --- | --- | --- |
| **97** | |  | |  | |  | |  | |  | |  | |  | |  | |  | |  | |  | | C228T | |  |  |  |
| **98** | |  | |  | |  | |  | |  | |  | |  | |  | |  | |  | |  | | C228T | |  |  |  |
| **99** | |  | |  | |  | |  | |  | |  | |  | |  | |  | |  | |  | | C228T | | R248Q  41.38% |  |  |
| **100** | | V600E  5.65% | | H66R  5.12% | |  | |  | |  | |  | |  | |  | |  | |  | |  | | C228T | | Q331*  7.41% |  |  |
|  | |  | |  | |  | |  | |  | |  | |  | |  | |  | |  | |  | |  | | T125A  6.21% |  |  |
| **101** | |  | |  | |  | |  | |  | |  | |  | |  | |  | |  | |  | | C228T | |  |  |  |
| **102** | |  | | E88G  5.4% | |  | |  | |  | |  | |  | |  | |  | |  | |  | | C228T | |  |  |  |
| **103** | |  | |  | |  | |  | |  | |  | |  | |  | |  | |  | |  | | C228T | |  |  |  |
| **104** | |  | | E88G  7.60% | |  | |  | |  | |  | |  | |  | |  | |  | |  | |  | | T125A  5.84% |  |  |
| **105** | |  | |  | |  | |  | |  | |  | |  | |  | |  | |  | |  | | C228T | |  |  |  |
| **106** | |  | | E88G  10.44% | |  | |  | |  | |  | |  | |  | |  | | S576fs  23.88% | |  | |  | | E294fs  40.73% |  |  |
|  | |  | | N84G  5.32% | |  | |  | |  | |  | |  | |  | |  | |  | |  | |  | |  |  |  |
| **107** | |  | |  | |  | |  | |  | | H27L  8.33% | |  | |  | |  | |  | |  | |  | | S183*  24.36% |  |  |
| **109** | |  | |  | |  | |  | |  | |  | |  | |  | |  | |  | |  | | C228T | |  |  |  |
| **110** | |  | | E88G  5.42% | |  | |  | |  | |  | |  | |  | |  | |  | |  | | C250T | |  |  |  |
| **111** | |  | |  | |  | |  | |  | |  | |  | |  | |  | |  | |  | |  | | R209fs  52.79% |  |  |
| **112** | |  | |  | |  | |  | |  | |  | |  | |  | |  | |  | |  | |  | | E285L  23.43% |  |  |
|  | |  | |  | |  | |  | |  | |  | |  | |  | |  | |  | |  | |  | |  |  |  |
| **Sample** | ***BRAF*** | | ***CDKN2A*** | | ***EGFR*** | | ***ERRB2*** | | ***HRAS*** | | ***KRAS*** | | ***NRAS*** | | ***PDGFRA*** | | ***PIK3CA*** | | ***RB1*** | | ***RET*** | | ***TERT*** | | ***TP53*** | | ***TSC2*** | |
| **113** |  | | E88G  14.84% | |  | |  | |  | |  | |  | |  | |  | |  | |  | |  | | C275fs  42.70% | |  | |
|  |  |  | L65P  5.63% | |  |  |  |  |  |  |  |  |  |  |  |  |  |  |  |  |  |  |  |  | T125A  7.08% | |  |  |
| **114** |  | |  | |  | |  | |  | | G12R  49.22% | |  | |  | | Q545L  32.43% | |  | |  | | C228T | | R280T  22.78% | |  | |
| **115** |  | |  | |  | | L787P  7.55% | | Q61R  9.64% | |  | |  | |  | |  | |  | |  | |  | | L299P  6.47% | |  | |
|  |  |  |  |  |  |  |  |  |  |  |  |  |  |  |  |  |  |  |  |  |  |  |  |  | G245V  8.99% | |  |  |
| **116** |  | |  | |  | |  | |  | |  | |  | |  | |  | |  | |  | |  | |  | |  | |
| **117** |  | | E88G  10.76% | |  | |  | |  | |  | |  | |  | |  | |  | |  | | C228T | | R282W  28.4% | |  | |
|  |  |  |  |  |  |  |  |  |  |  |  |  |  |  |  |  |  |  |  |  |  |  |  |  | V272L  22.49% | |  |  |
| **118** |  | | E88G  8.12% | |  | |  | |  | |  | |  | |  | |  | |  | |  | | C228T | |  | |  | |
| **119** |  | | E88G  6.21% | |  | |  | |  | |  | |  | |  | |  | |  | |  | |  | | C135F  76.47% | |  | |
| **120** |  | |  | |  | |  | |  | | G12R  60.74% | |  | |  | | H1047R  29.94% | |  | |  | | C228T | | E285K  33.96% | |  | |
| **121** |  | |  | |  | |  | |  | |  | |  | |  | |  | |  | |  | | C228T | |  | |  | |

Supplemental Table 1: Mutations in the analysed genes 118 ATC primary tumour samples and the relative prevalence of each mutation in %.
